# The superiority of multi-trait models with genotype-by-environment interactions in a limited number of environments for genomic prediction in pigs

**DOI:** 10.1186/s40104-020-00493-8

**Published:** 2020-08-19

**Authors:** Hailiang Song, Qin Zhang, Xiangdong Ding

**Affiliations:** 1 National Engineering Laboratory for Animal Breeding, Laboratory of Animal Genetics, Breeding and Reproduction, Ministry of Agriculture, College of Animal Science and Technology, China Agricultural University, Beijing, 100193 China; 2 Shandong Provincial Key Laboratory of Animal Biotechnology and Disease Control and Prevention, Shandong Agricultural University, Taian, 271001 China

**Keywords:** Combined population, Genotype-by-environment interaction, Linkage disequilibrium, Multi-trait model, Pig

## Abstract

**Background:**

Different production systems and climates could lead to genotype-by-environment (G × E) interactions between populations, and the inclusion of G × E interactions is becoming essential in breeding decisions. The objective of this study was to investigate the performance of multi-trait models in genomic prediction in a limited number of environments with G × E interactions.

**Results:**

In total, 2,688 and 1,384 individuals with growth and reproduction phenotypes, respectively, from two Yorkshire pig populations with similar genetic backgrounds were genotyped with the PorcineSNP80 panel. Single- and multi-trait models with genomic best linear unbiased prediction (GBLUP) and BayesC *π* were implemented to investigate their genomic prediction abilities with 20 replicates of five-fold cross-validation. Our results regarding between-environment genetic correlations of growth and reproductive traits (ranging from 0.618 to 0.723) indicated the existence of G × E interactions between these two Yorkshire pig populations. For single-trait models, genomic prediction with GBLUP was only 1.1% more accurate on average in the combined population than in single populations, and no significant improvements were obtained by BayesC *π* for most traits. In addition, single-trait models with either GBLUP or BayesC *π* produced greater bias for the combined population than for single populations. However, multi-trait models with GBLUP and BayesC *π* better accommodated G × E interactions, yielding 2.2% – 3.8% and 1.0% – 2.5% higher prediction accuracies for growth and reproductive traits, respectively, compared to those for single-trait models of single populations and the combined population. The multi-trait models also yielded lower bias and larger gains in the case of a small reference population. The smaller improvement in prediction accuracy and larger bias obtained by the single-trait models in the combined population was mainly due to the low consistency of linkage disequilibrium between the two populations, which also caused the BayesC *π* method to always produce the largest standard error in marker effect estimation for the combined population.

**Conclusions:**

In conclusion, our findings confirmed that directly combining populations to enlarge the reference population is not efficient in improving the accuracy of genomic prediction in the presence of G × E interactions, while multi-trait models perform better in a limited number of environments with G × E interactions.

## Background

Genomic selection [1], which relies on linkage disequilibrium (LD) between single nucleotide polymorphisms (SNPs) and causative variants, has become a useful tool in animal breeding [2] and plant breeding [3]. Generally, the accuracy of genomic selection increases as the number of animals in the reference population increases [4]. For small reference populations, combining populations of the same breed or related breeds reportedly increases the accuracy of genomic selection in cattle and pig [2, 5–8]. However, genomic prediction using combined populations has not shown a clear advantage over genomic prediction using single populations, possibly because the LD between SNPs and causative variants is not sufficiently consistent between populations [5–7]. The failure to consider genotype-by-environment (G × E) interactions between populations is another important reason. Thus, exploiting G × E interactions in combined populations could be an attractive and meaningful approach for increasing the accuracy of genomic prediction.

A G × E interaction is defined as different genotypes reacting unequally to environmental changes [9], and ignoring possible G × E interactions could lead to a reduction in genetic gains. Two models are widely used to detect G × E interactions. One is a multi-trait model, which assumes that the phenotypic expressions of a trait in various environments are different traits [10]. In this case, the genetic correlation between traits in different environments is used as the indicator of a G × E interaction [9, 11]. The other model is the reaction norm model [12], which models the trajectory of animal performance as a function of an environmental gradient. However, in a small number of environments, e.g., two or three environments, the reaction norm model captures only part of the G × E interaction due to the limited amount of environmental variation. Furthermore, it is difficult to define a suitable environmental covariate in the reaction norm model [12, 13].

With the development of genomic selection in pig breeding, more farms could be included to enlarge the reference population size in China, and joint genomic evaluation across countries or breeding organizations is expected. In dairy cattle, G × E interactions are usually ignored in combined-population genomic prediction; for example, Zhou et al. [14] reported that the consistency of LD was very high (0.97) between Chinese and Nordic Holsteins, indicating a high level of genetic similarity between the two populations. Therefore, it is necessary to consider the influence of G × E interactions, as the consistency of LD between pig populations is relatively low compared to that in dairy cattle. The objectives of this study were to evaluate G × E interactions between two Yorkshire pig populations with similar genetic backgrounds and to investigate the performance of multi-trait models in genomic prediction in a limited number of environments with G × E interactions.

## Methods

### Ethics statement

The whole procedure for collecting pig blood samples was carried out in strict accordance with the protocol approved by the Animal Welfare Committee of China Agricultural University (Permit Number: DK996).

### Population and phenotypes

Yorkshire pig populations were sampled from two breeding farms in the north (Beijing) and south (Fujian Province) of China, designated Beijing and Fujian, respectively, for convenience. The pigs in the Beijing and Fujian populations are American Yorkshire progeny with the same genetic background but no pedigree links. Phenotypic records of reproductive traits, namely, the number of piglets born alive (NBA) and the total number of piglets born (TNB), and growth traits, namely, days to 100 kg (AGE) and backfat thickness at 100 kg (BFT), were examined. The measurements of AGE and BFT were reported in Song et al. [7]. The phenotypic information is presented in Table 1. For the growth traits, phenotypic information from 28,827 and 13,860 pigs born in 2008–2017 and 2007–2018, respectively, in the Beijing and Fujian populations was available. For the reproductive traits, the farrow records of 5,968 and 3,115 pigs born in 2007–2017 and 2007–2018, respectively, in Beijing and Fujian were obtained.

**Table 1:** Summary statistics for the two Yorkshire pig populations, including the numbers of genotyped animals and estimates of heritability (*h*^2^)

Population^a^	Trait^b^	N-obs^c^	Max	Min	SD	Genotyped animals	*h*^2^ (SE)
Beijing	AGE	28,827	210.98	124	13.86	1732	0.33(0.01)
	BFT	28,827	30.74	5.03	2.43	1732	0.34 (0.01)
	NBA	5,968/20,005	21	0	3.13	923	0.07 (0.01)
	TNB	5,968/20,005	25	0	3.09	923	0.08 (0.01)
Fujian	AGE	13,860	206.31	119.9	10.27	956	0.42 (0.02)
	BFT	13,860	28.62	4.09	3.64	956	0.44 (0.02)
	NBA	3,115/11,731	22	0	2.88	461	0.09 (0.02)
	TNB	3,115/11,731	22	1	2.98	461	0.11 (0.02)

^a^ Yorkshire pig populations from Beijing and Fujian with similar genetic backgrounds but located in the north and south of China

^b^ AGE: days to 100 kg; BFT: backfat thickness at 100 kg; NBA: number of piglets born alive; TNB: total number of piglets born

^c^ N-obs: number of individuals/observations

### Construction of corrected phenotypes

In pig genomic prediction analyses, corrected phenotypes derived from pedigree-based estimated breeding values (EBVs) are usually used as response variables. Conventional EBVs and genetic parameters were estimated based on a repeatability model of reproductive traits implemented separately in each population. In the model, the fixed effects included herd, year and season, and the random effects included the additive genetic, random residual and permanent environmental effects. For the growth traits, a bivariate animal model was implemented, and the fixed effects included herd, year, season and sex. In addition to the additive genetic effect of each individual and the random residual effect, litter effect was also taken into account as a random effect. A total of 31,529 and 32,175 animals were traced back to construct a pedigree relationship matrix for the Beijing and Fujian populations, respectively. Afterwards, corrected phenotypic values (*y*_*c*_) for reproductive traits were calculated as the EBV plus the average of estimated residuals over parities for a sow, and *y*_*c*_ values for growth traits were computed as the EBV plus the estimated residual for each individual in each population. EBVs and genetic parameters were computed using the DMUAI procedure in DMU software [15].

### Genotype data and quality control

Genomic DNA was extracted from blood samples using a TIANamp Blood DNA Kit DP348 (Tiangen, Beijing, China). Genotyping was performed using the PorcineSNP80 BeadChip (Illumina, San Diego, CA, USA), which includes 68,528 SNPs distributed across the entire pig genome. The number of genotyped animals for each trait is presented in Table 1.

Missing genotypes for SNPs with known chromosomal positions were imputed by BEAGLE with default parameter settings [16], and those for SNPs with unknown positions were discarded. Based on the imputed dataset, SNPs were excluded from the analysis if the minor allele frequency was less than 0.05, the call frequency score was less than 0.90, or the genotype frequencies deviated from Hardy-Weinberg equilibrium with a *P*-value lower than 10^− 7^. The animals with a call rate less than 0.90 or with an EBV reliability less than 0.3 were removed. After quality control, 2,688 and 1,384 genotyped individuals remained for the growth and reproductive traits, respectively, and 56,445 SNPs were ultimately used.

### Principal component analysis

Principal component analysis (PCA) was performed on the **G** matrix using GCTA software [17]. This resulted in a matrix of eigenvectors in descending order that represented principal components (PCs), where PC1 had the largest eigenvalue. The overall structuring of genetic variation was visualized in a scatterplot of the top few PCs.

### Measure of linkage disequilibrium

LD between a pair of SNPs was measured as ${r}_{LD}^2$ and *r*_*LD*_ [18], and ${r}_{LD}^2$ was calculated as follows:

$$ {r}_{LD}^2=\frac{{\left(f(AB)-f(A)f(B)\right)}^2}{f(A)f(a)f(B)f(b)}, $$

where *f*(*AB*), *f*(*A*), *f*(*a*), *f*(*B*) and *f*(*b*) are the observed frequencies of haplotype AB and alleles A, a, B and b, respectively. The consistency of LD in the two populations was measured by the correlation of *r*_*LD*_ values of adjacent marker pairs on each autosome between the two populations.

### Model

Four methods, namely, single-trait genomic BLUP (GBLUP), multi-trait GBLUP, single-trait BayesC *π* and multi-trait BayesC *π*, were implemented to predict the genomic EBV (GEBV) for each genotyped individual.

#### Single-trait GBLUP (ST-GBLUP) model

The ST-GBLUP [19] model was used to predict the GEBVs of all genotyped individuals.

$$ \boldsymbol{y}=\mathbf{1}\boldsymbol{u}+\boldsymbol{Za}+\boldsymbol{e}, $$

where ***y*** is the vector of corrected phenotypic values; ***u*** is the overall mean; **1** is a vector of 1; and ***a*** is the vector of genomic breeding values, following a normal distribution N(0, **G** *σ*_*a*_^2^), in which *σ*_*a*_^2^ is the variance of the addictive genetic effect and **G** is the marker-based genomic relationship matrix [19]. ***e*** is the vector of random errors, following a normal distribution N(0, **I** *σ*_*e*_^2^), in which *σ*_*e*_^2^ is the residual variance.

#### Multi-trait GBLUP (MT-GBLUP) model

The MT-GBLUP model was defined as.

$$ \left[\begin{array}{c}{\boldsymbol{y}}_{\mathbf{1}}\\ {}{\boldsymbol{y}}_{\mathbf{2}}\end{array}\right]=\left[\begin{array}{cc}{\mathbf{I}}_{\mathbf{1}}& \mathbf{0}\\ {}\mathbf{0}& {\mathbf{I}}_{\mathbf{2}}\end{array}\right]\left[\begin{array}{c}{u}_1\\ {}{u}_2\end{array}\right]+\left[\begin{array}{cc}{\boldsymbol{Z}}_{\mathbf{1}}& \mathbf{0}\\ {}\mathbf{0}& {\boldsymbol{Z}}_{\mathbf{2}}\end{array}\right]\left[\begin{array}{c}{\boldsymbol{g}}_{\mathbf{1}}\\ {}{\boldsymbol{g}}_{\mathbf{2}}\end{array}\right]+\left[\begin{array}{c}{\boldsymbol{e}}_{\mathbf{1}}\\ {}{\boldsymbol{e}}_{\mathbf{2}}\end{array}\right], $$

where $$ \left[\begin{array}{c}{\boldsymbol{y}}_{\mathbf{1}}\\ {}{\boldsymbol{y}}_{\mathbf{2}}\end{array}\right] $$ is the vector of observed values for traits I and II (corrected phenotypic values of the same trait in different populations); ***I***_**1**_ and ***I***_**2**_ are the identity matrices; $$ \left[\begin{array}{c}{u}_1\\ {}{u}_2\end{array}\right] $$ is the vector of intercepts for traits I and II; $$ \left[\begin{array}{c}{\boldsymbol{g}}_{\mathbf{1}}\\ {}{\boldsymbol{g}}_{\mathbf{2}}\end{array}\right] $$ is the vector of additive genetic effects for the two traits, following a normal distribution N(**0**, **G** ⊗ **M**), where **M**= $$ \left[\begin{array}{cc}{\sigma}_{g1}^2& {\sigma}_{g12}^2\\ {}{\sigma}_{g12}^2& {\sigma}_{g2}^2\end{array}\right] $$ is the variance and covariance matrix of the genomic breeding values of the two traits; ***Z***_**1**_ and ***Z***_**2**_ are the incidence matrices associating ***g***_**1**_ and ***g***_**2**_ with ***y***_**1**_ and ***y***_**2**_; and $$ \left[\begin{array}{c}{\boldsymbol{e}}_{\mathbf{1}}\\ {}{\boldsymbol{e}}_{\mathbf{2}}\end{array}\right] $$ is the vector of random errors with a distribution of N(**0**, **I** ⊗ **R**), where **I** is the identity matrix and **R** = $$ \left[\begin{array}{cc}{\sigma}_{e1}^2& {\sigma}_{e12}^2\\ {}{\sigma}_{e12}^2& {\sigma}_{e2}^2\end{array}\right] $$ is the residual variance and covariance matrix of the two traits. The genetic correlation between two traits was calculated as $\frac{\sigma_{g12}^2}{\sqrt{\sigma_{g1}^2{\sigma}_{g2}^2}}$.

#### Single-trait BayesC π (ST-BayesC π) model

In ST-BayesC *π* [20], marker effects on phenotypic traits were sampled from a mixture of null and normal distributions,

$$ {\boldsymbol{y}}_i=\boldsymbol{\mu} +\sum \limits_j^p{m}_{ij}{\alpha}_j+{\boldsymbol{e}}_i $$

$$ \left({\alpha}_j|\pi, {\sigma}_a^2\right)\left\{\begin{array}{c}\sim N\left(0,{\sigma}_a^2\right)\  probability\left(1-\pi \right)\\ {}0\  probability\ \pi \end{array}\right. $$

where ***y***_*i*_ is a vector of phenotypes, ***μ*** is a vector of overall means, *m*_*ij*_ is the genotype covariate at locus *j* for individual *i* (coded as 0, 1 and 2), *p* is the number of genotyped loci, *α*_*j*_ is a vector of allele substitution effects, and ***e***_*i*_ is a vector of random residuals for individual *i* with a distribution N(0, ${\sigma}_e^2$). The markers in the model shared a common variance ${\sigma}_a^2$. The prior for the allele substitution effects of each molecular marker *α*_*j*_ depends on the variance ${\sigma}_a^2$ and the probability *π* that markers do not have a genetic effect.

#### Multi-trait BayesC π (MT-BayesC π) model

In MT-BayesC *π*, where each locus can have an effect on any combination of traits [21], the prior for *α*_*jk*_**,** the allele substitution or marker effect on trait *k* for locus *j*, is a mixture with a point mass at zero and univariate normal distribution conditional on ${\sigma}_k^2$:

$$ {\boldsymbol{y}}_i=\boldsymbol{\mu} +\sum \limits_j^p{m}_{ij}{\boldsymbol{\alpha}}_j+{\boldsymbol{e}}_i $$

$$ \left({\alpha}_{jk}|{\pi}_k,{\sigma}_k^2\right)\left\{\begin{array}{c}\sim N\left(0,{\sigma}_k^2\right)\  probability\left(1-{\pi}_k\right)\\ {}0\  probability\ {\pi}_k\end{array}\right. $$

where *m*_*ij*_, *j* and *p* are the same as in ST-BayesC *π*. ***y***_*i*_ is a vector of phenotypes of *k* traits for individual *i* (corrected phenotypic values of the same trait in different populations), ***μ*** is a vector of overall means for *k* traits, and ***α***_*j*_ is a vector of marker allele substitution effects on *k* traits for locus *j*. The residuals, ***e***_*i*_, are a priori assumed to be independently and identically distributed multivariate normal vectors with a null mean and covariance matrix **R** (**R** is the same as described for the multi-trait GBLUP model), which, in turn, is assumed to have an inverse Wishart prior distribution, ${\boldsymbol{W}}_{\boldsymbol{t}}^{-\mathbf{1}}\left({\boldsymbol{S}}_{\boldsymbol{e}},{\boldsymbol{v}}_{\boldsymbol{e}}\right)$**.** The covariance between effects for trait *k* and *k*′ at the same locus, i.e., *α*_*jk*_ and *α*_*jk*′_ is

$$ \mathit{\operatorname{cov}}\left({\alpha}_{jk},{\alpha}_{j{k}^{\prime }}|{\sigma}_{kk\prime}\right)\left\{\begin{array}{c}{\sigma}_{k{k}^{\prime }}\  if\ both\ {\alpha}_{jk}\ne 0\  and\ {\sigma}_{k{k}^{\prime }}\ne 0\Big)\\ {}0\  otherwise\ \end{array}\right.. $$

Imputation of missing phenotypic data was implemented in each Markov chain Monte Carlo (MCMC) iteration in MT-BayesC *π*.

For the ST-BayesC *π* and MT-BayesC *π* methods, the MCMC chain was run for 20,000 cycles, and the first 10,000 cycles were discarded as burn-in. Every 10^th^ sample of the remaining 10,000 iterations was saved to estimate SNP effects and the variance components. The Julia package JWAS was used for BayesC *π* analyses [22], and the DMUAI procedure, implemented in DMU software, was used for GBLUP analyses.

### Cross-validation and prediction accuracy

In this study, the accuracy and unbiasedness of prediction were obtained through 5-fold cross-validation (CV) for the Beijing and Fujian populations, where the Beijing (Fujian) population was randomly split into 5 folds, predicting one fold based on the other 4 folds, the combined population was divided into 4 folds and another population, or a multi-trait model was used in which the values of the same trait in 4 folds and another population were considered different traits. In each round of CV, phenotypes from one fold (validation population) were removed from the dataset, and the remaining folds (reference population) were used to predict the future performance of animals in the validation population. This 5-fold CV was replicated 20 times, and the results are presented as the mean and standard deviation for the 20 replicates. Here, the single-trait models (ST-GBLUP and ST-BayesC *π*) based on single populations and the combined population were termed ST-GBLUP(BayesC *π*)_single and ST-GBLUP(BayesC *π*)_combined, respectively. For ST-GBLUP(BayesC *π*)_single, GBLUP(BayesC *π*)_combined and MT-GBLUP(BayesC *π*), the validation population was the same for the four approaches in each replicate of 5-fold CV. The accuracy of genomic prediction was evaluated as *r*(*y*_*c*_, GEBV), the correlation between GEBVs and corrected phenotypic values *y*_*c*_ in the validation population. In addition, *b*(*y*_*c*_, GEBV), the regression of *y*_*c*_ on GEBVs, was also calculated to assess the possible inflation or unbiasedness of predictions. A two-tailed paired t-test was used to compare prediction accuracy between a pair of scenarios.

## Results

### Population structure and genetic parameters

**Fig. 1:**
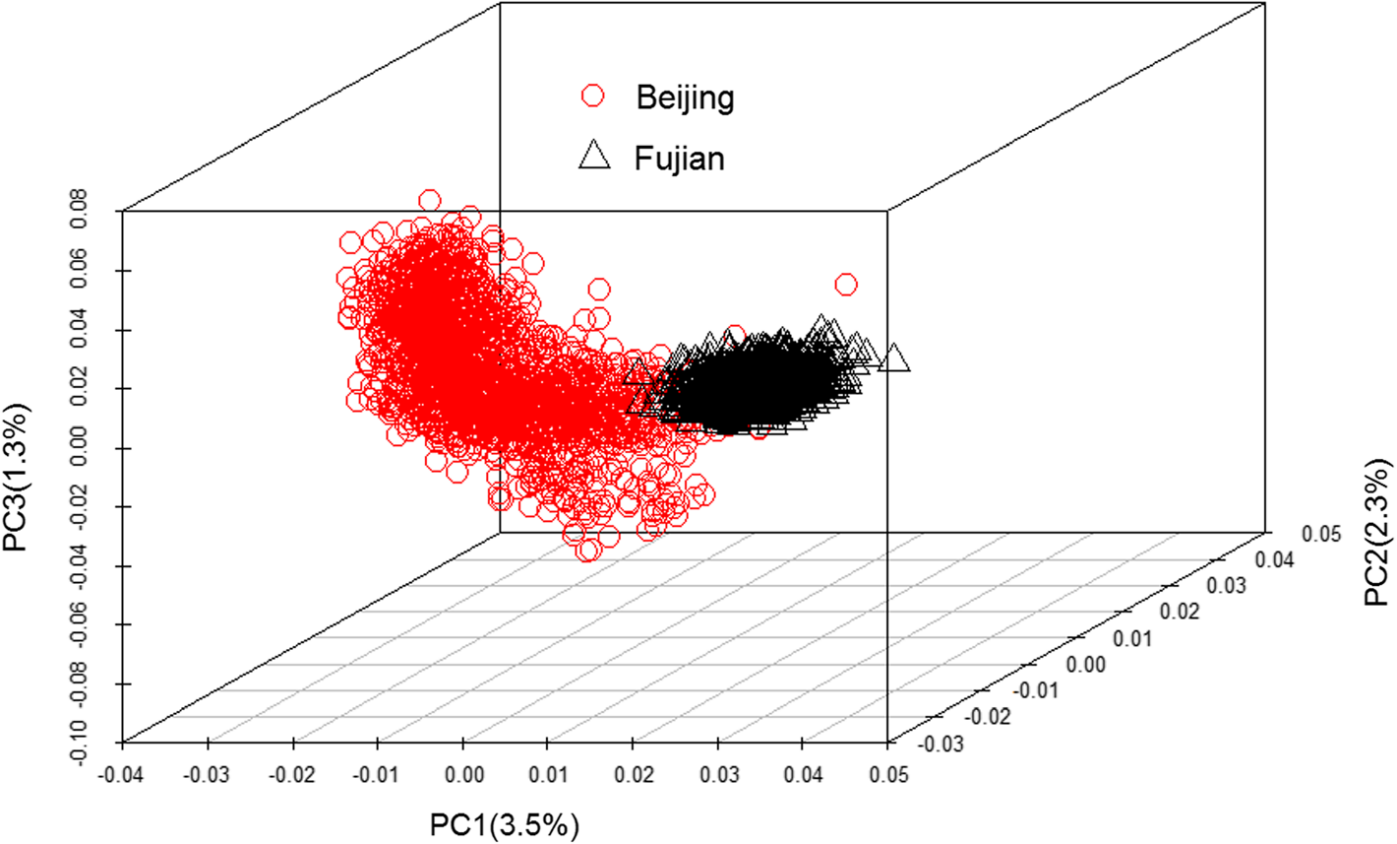
Principal component analysis (PCA) of two Yorkshire pig populations. Beijing and Fujian represent two Yorkshire pig populations from two Chinese pig breeding farms; PC1 (3.5%) = first principal component (variance explained by PC1 = 3.5%); PC2 (2.3%) = second principal component (variance explained by PC2 = 2.3%); PC3 (1.3%) = third principal component (variance explained by PC3 = 1.3%)

To assess the population structure of the two Yorkshire pig populations, PCA was performed. Figure 1 illustrates that the genetic backgrounds of the Beijing and Fujian populations were similar, and both populations were composed of American Yorkshire progeny. In addition, the Yorkshire population (874 pigs) with a British origin was selected for PCA, which also showed that Beijing and Fujian had similar genetic backgrounds (Additional file: Fig. S1). However, no pedigree connection between these populations was detected according to their pedigree records due to a lack of genetic exchange.

LD between a pair of SNPs was measured as ${r}_{LD}^2$ and *r*_*LD*_ in the Beijing and Fujian populations. The LD between adjacent markers in the Beijing and Fujian populations was also investigated, as shown in Table 2. The mean ${r}_{LD}^2$ of adjacent SNP pairs within a chromosome ranged from 0.52 to 0.63 in the Beijing population and from 0.51 to 0.63 in the Fujian population. The mean ${r}_{LD}^2$ across all chromosomes was 0.56 in both the Beijing and Fujian populations, also indicating the similar genetic backgrounds of the populations. However, the consistency of LD between the two populations was not high. The correlation of *r*_*LD*_ for adjacent SNPs between the two populations ranged from 0.47 to 0.60 across all chromosomes, with a mean of 0.55 at an average marker distance of 41 Kb, indicating the insufficient consistency in LD between these two pig populations.

**Table 2:** Distance and LD (${r}_{LD}^2$) of adjacent SNPs for each autosome (CHR)

CHR	Length, Mb	Number of SNPs	Mean distance, kb	Mean ${r}_{LD}^2$		Cor^a^
				Beijing^c^	Fujian^c^	
1	300.49	5,525	55.70	0.63	0.63	0.55
2	155.0	3,750	42.34	0.56	0.54	0.51
3	138.07	3,267	43.29	0.54	0.53	0.54
4	136.82	3,453	40.59	0.57	0.57	0.51
5	106.34	2,671	40.78	0.54	0.54	0.60
6	150.43	3,967	38.84	0.61	0.60	0.47
7	128.52	3,563	36.95	0.54	0.53	0.58
8	141.61	3191	45.46	0.57	0.56	0.56
9	146.54	3,533	42.48	0.55	0.57	0.58
10	75.40	2,522	30.63	0.55	0.53	0.51
11	83.61	2,105	40.69	0.52	0.51	0.50
12	60.64	2,247	27.65	0.57	0.56	0.60
13	208.48	4,026	53.04	0.59	0.60	0.58
14	146.71	3,756	40.01	0.59	0.59	0.57
15	150.35	3,208	48.01	0.58	0.59	0.52
16	82.85	2,154	39.41	0.53	0.52	0.51
17	66.13	1,938	34.96	0.58	0.58	0.53
18	58.37	1,569	38.12	0.54	0.52	0.55
Mean	2,336.36^b^	56,445^b^	41.05	0.56	0.56	0.55

^a^ Cor: the correlation of *r*_*LD*_ values of adjacent SNP pairs between two populations;

^b^ Sum over 18 autosomes

^c^ Yorkshire pig populations from Beijing and Fujian with similar genetic backgrounds

Estimates of heritability for the growth and reproductive traits in the two Yorkshire pig populations are shown in Table 1. In the Beijing population, the heritabilities of AGE and BFT were 0.33 and 0.34, respectively, with a standard error of 0.01. The heritabilities of AGE and BFT were 0.42 and 0.44, respectively, with a standard error of 0.02 in the Fujian population. For NBA and TNB, the estimated heritabilities were 0.07 and 0.08 with a standard error of 0.01 in the Beijing population and 0.09 and 0.11 with a standard error of 0.02 in the Fujian population.

### G × E interactions

**Fig. 2:**
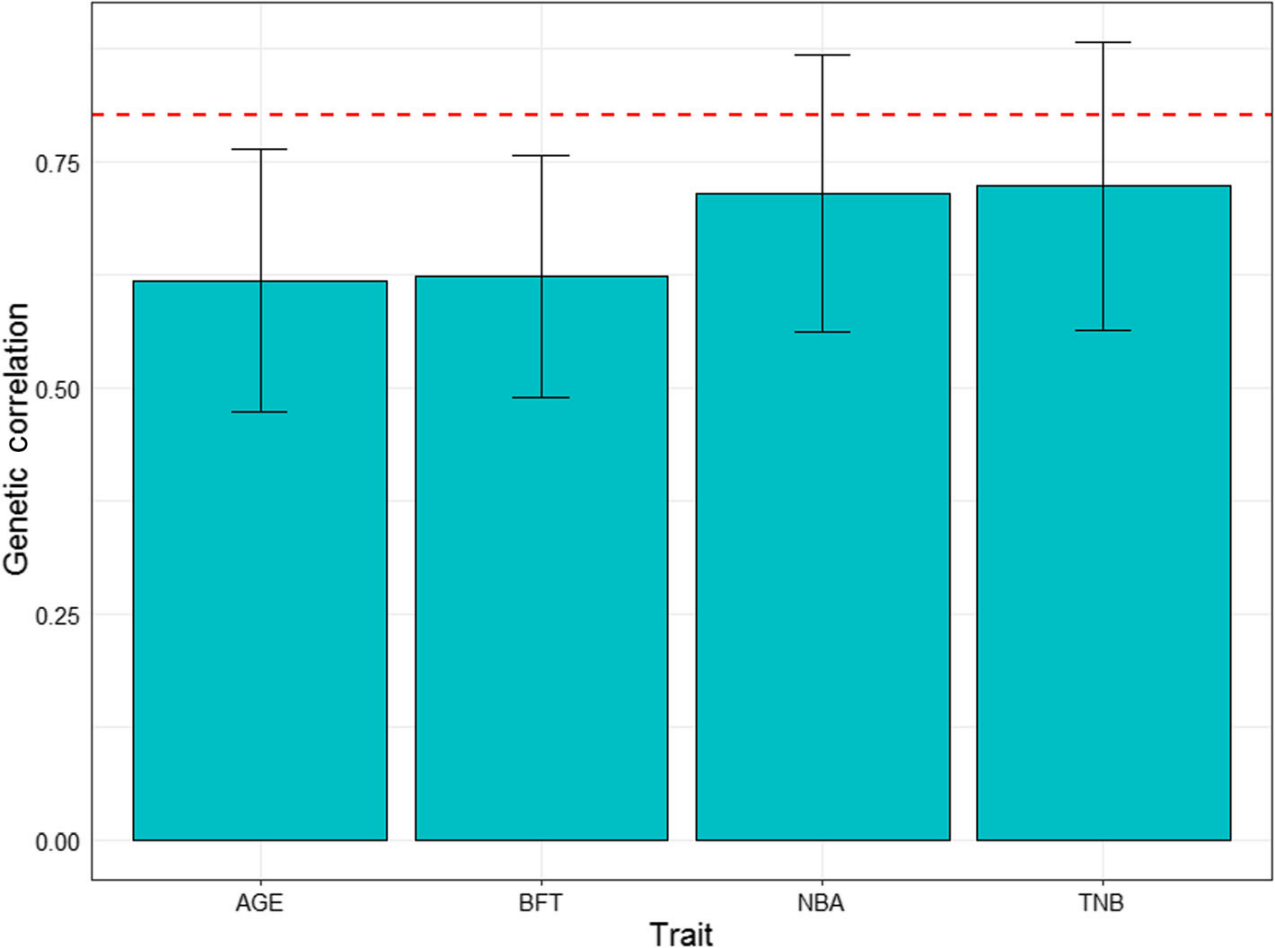
Genetic correlations between the two Yorkshire pig populations obtained using the multi-trait GBLUP method with all genotyped animals. AGE: days to 100 kg; BFT: backfat thickness at 100 kg; NBA: number of piglets born alive; TNB: total number of piglets born. The red line represents the threshold of 0.8. The error bar represents the standard error

Figure 2 shows the estimated genetic correlations between the Beijing and Fujian populations based on the MT-GBLUP model using all genotyped animals. For AGE and BFT, the estimates of genetic correlations were 0.618 and 0.623, respectively, with standard errors of 0.145 and 0.134. The genetic correlations of NBA and TNB were 0.714 and 0.723, respectively, with standard errors of 0.153 and 0.159. These genetic correlations indicated that G × E interactions most likely exist between the Beijing and Fujian populations, as Robertson [23] suggested that 0.80 was the threshold of biological importance for G × E interactions.

**Fig. 3:**
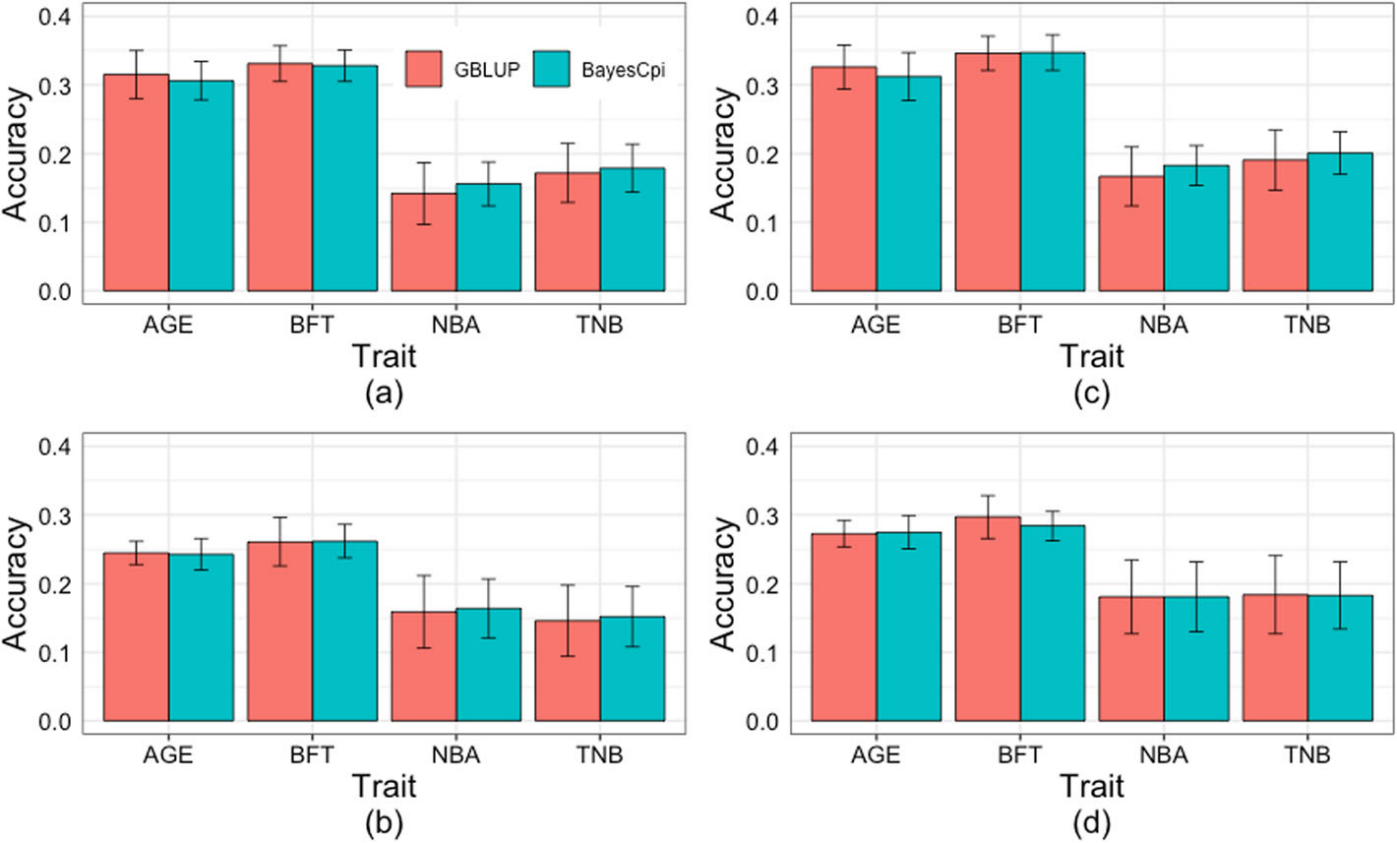
Comparison of genomic prediction accuracies of GBLUP and BayesC *π* methods when implementing a single-trait model in single populations and a multi-trait model. **a** Predicting pigs from the Beijing population using a single-trait model; **b** predicting pigs from the Fujian population using a single-trait model; **c** predicting pigs from the Beijing population using a multi-trait model; **d** predicting pigs from the Fujian population using a multi-trait model. AGE: days to 100 kg; BFT: backfat thickness at 100 kg; NBA: number of piglets born alive; TNB: total number of piglets born; BayesCpi: BayesC *π*

Tables 3 and 4 and Fig. 3 demonstrate the accuracies of genomic prediction for growth and reproductive traits achieved by applying single-trait and multi-trait models with GBLUP and BayesC *π*. Generally, in the same scenario, the prediction accuracies of each method were the largest for BFT, as it had the highest estimated heritability among the four traits. Lower prediction accuracies were obtained for two reproductive traits, NBA and TNB, due to their low heritabilities. For the two pig populations, higher accuracies for growth traits were acquired for the Beijing population, as it had a much larger reference population than the Fujian population (Table 1), while their small reference populations for reproductive traits resulted in comparably low accuracies of genomic prediction. Tables 3 and 4 and Fig. 3 further show the superiority of the multi-trait model for genomic prediction when dealing with G × E interactions, as discussed below.

**Table 3:** Accuracy and unbiasedness of genomic prediction of growth and reproductive traits performed with the GBLUP method as assessed with 20 replicates of five-fold CV

Population^1^	Method^2^	Measurement^3^	AGE^4^	BFT^4^	NBA^4^	TNB^4^
Beijing	ST-GBLUP_single	Accuracy	0.315 ± 0.035^a^	0.331 ± 0.026^a^	0.142 ± 0.045^a^	0.172 ± 0.043^a^
		Unbiasedness	1.001 ± 0.203	1.126 ± 0.254	1.220 ± 0.413	1.146 ± 0.411
	ST-GBLUP_combined	Accuracy	0.315 ± 0.037^a^	0.334 ± 0.029^a^	0.153 ± 0.052^b^	0.189 ± 0.052^b^
		Unbiasedness	1.119 ± 0.222	0.827 ± 0.249	0.698 ± 0.360	0.799 ± 0.284
	MT-GBLUP	Accuracy	0.326 ± 0.032^b^	0.346 ± 0.025^b^	0.167 ± 0.043^c^	0.191 ± 0.044^b^
		Unbiasedness	0.994 ± 0.196	1.083 ± 0.225	1.164 ± 0.362	1.112 ± 0.278
Fujian	ST-GBLUP_single	Accuracy	0.245 ± 0.017^a^	0.261 ± 0.035^a^	0.159 ± 0.053^a^	0.146 ± 0.052^a^
		Unbiasedness	1.047 ± 0.267	1.046 ± 0.263	1.047 ± 0.442	1.050 ± 0.421
	ST-GBLUP_combined	Accuracy	0.255 ± 0.023^b^	0.272 ± 0.043^b^	0.171 ± 0.062^b^	0.173 ± 0.055^b^
		Unbiasedness	1.352 ± 0.303	1.274 ± 0.271	1.352 ± 0.454	1.191 ± 0.465
	MT-GBLUP	Accuracy	0.273 ± 0.019^c^	0.297 ± 0.031^c^	0.181 ± 0.054^c^	0.184 ± 0.057^c^
		Unbiasedness	1.024 ± 0.243	1.013 ± 0.232	1.032 ± 0.433	1.046 ± 0.397

^1^ Yorkshire pig populations from Beijing and Fujian with similar genetic backgrounds

^2^ ST-GBLUP_single: single-trait GBLUP model with a single population as the reference population; ST-GBLUP_combined: single-trait GBLUP model two populations combined as the reference population; MT-GBLUP: multi-trait GBLUP model in which values of the same trait in different populations were considered different traits

^3^ Accuracy: the correlation between GEBV and corrected phenotypic values in the validation population; Unbiasedness: the regression of corrected phenotypic values on GEBVs

^4^ AGE: days to 100 kg; BFT: backfat thickness at 100 kg; NBA: number of piglets born alive; TNB: total number of piglets born

^a, b, c^ Values with different superscript letters significantly differ (*P* < 0.05)

**Table 4:** Accuracy and unbiasedness of genomic prediction of growth and reproductive traits performed with the BayesC *π* method as assessed with 20 replicates of five-fold CV

Population^1^	Method^2^	Measurement^3^	AGE^4^	BFT^4^	NBA^4^	TNB^4^
Beijing	ST-BayesC *π* _single	Accuracy	0.306 ± 0.028^a^	0.328 ± 0.023^a^	0.156 ± 0.032^a^	0.179 ± 0.035^a^
		Unbiasedness	1.218 ± 0.278	1.013 ± 0.212	1.182 ± 0.343	1.112 ± 0.302
	ST-BayesC *π* _combined	Accuracy	0.304 ± 0.032^a^	0.328 ± 0.038^a^	0.154 ± 0.035^a^	0.180 ± 0.034^a^
		Unbiasedness	1.287 ± 0.265	0.853 ± 0.274	1.152 ± 0.362	1.124 ± 0.293
	MT-BayesC *π*	Accuracy	0.312 ± 0.035^b^	0.347 ± 0.026^b^	0.183 ± 0.029^b^	0.201 ± 0.031^b^
		Unbiasedness	1.160 ± 0.217	1.056 ± 0.224	1.065 ± 0.236	0.992 ± 0.272
Fujian	ST-BayesC *π* _single	Accuracy	0.243 ± 0.023^a^	0.262 ± 0.024^a^	0.164 ± 0.043^a^	0.152 ± 0.044^a^
		Unbiasedness	1.052 ± 0.263	1.032 ± 0.232	1.115 ± 0.321	1.076 ± 0.362
	ST-BayesC *π* _combined	Accuracy	0.244 ± 0.025^a^	0.264 ± 0.028^a^	0.165 ± 0.047^a^	0.154 ± 0.047^a^
		Unbiasedness	0.874 ± 0.134	1.330 ± 0.254	1.245 ± 0.308	1.222 ± 0.353
	MT-BayesC *π*	Accuracy	0.275 ± 0.024^b^	0.284 ± 0.021^b^	0.181 ± 0.051^b^	0.183 ± 0.049^b^
		Unbiasedness	1.034 ± 0.197	1.088 ± 0.256	1.143 ± 0.363	1.059 ± 0.345

^1^ Yorkshire pig populations from Beijing and Fujian with similar genetic backgrounds

^2^ ST-GBLUP_single: single-trait GBLUP model with a single population as the reference population; ST-GBLUP_combined: single-trait GBLUP model two populations combined as the reference population; MT-GBLUP: multi-trait GBLUP model in which values of the same trait in different populations were considered different traits

^3^ Accuracy: the correlation between GEBVs and corrected phenotypic values in the validation population; Unbiasedness: the regression of corrected phenotypic values on GEBVs

^4^ AGE: days to 100 kg; BFT: backfat thickness at 100 kg; NBA: number of piglets born alive; TNB: total number of piglets born

^a, b^ Values with different superscript letters significantly differ (*P* < 0.05)

### Comparison of the combined population with the single populations

Table 3 presents the accuracy of genomic prediction for growth and reproductive traits achieved by applying the GBLUP method, as measured by 20 replicates of five-fold CV. For the Beijing population, the prediction accuracies obtained with ST-GBLUP_single were 0.315 and 0.331 for AGE and BFT and 0.142 and 0.172 for NBA and TNB, respectively. When ST-GBLUP_combined was used, the genomic prediction accuracies were improved by 1.1% and 1.7% for NBA and TNB, respectively, compared with those obtained with ST-GBLUP_single, while there were no significant improvements for the growth traits AGE and BFT. In contrast, for the Fujian population, the genomic prediction accuracies for both growth and reproductive traits increased when the single populations were combined to enlarge the reference population. On average, ST_GBLUP_combined yielded 1.1% and 2.0% higher accuracies than ST-GBLUP_single for the growth and reproductive traits, respectively. Obviously, the gains in accuracy obtained for the Fujian population were larger.

However, the single-trait model with the BayesC *π* method did not yield higher prediction accuracies for the combined population compared with the single populations for most traits, even though the reference population was larger. As shown in Table 4, when predicting the traits of pigs from the Beijing population, the prediction accuracies obtained with ST-BayesC *π* _single were 0.306, 0.328, 0.156 and 0.179 for AGE, BFT, NBA and TNB, respectively. ST-BayesC *π* _combined produced almost the same accuracies as ST-BayesC *π* _single in all scenarios, and the accuracy obtained by ST-BayesC *π* _combined was even slightly lower in some cases; e.g., the prediction accuracies of ST-BayesC *π* _single and ST-BayesC *π* _combined for NBA were 0.156 and 0.154, respectively. A trend of similar accuracies for ST-BayesC *π* between the combined and single populations was also found in the Fujian population, as demonstrated in Table 4. The average prediction accuracies obtained with ST-BayesC *π* _single and ST-BayesC *π* _combined were almost the same for the growth traits and reproductive traits.

The unbiasedness of genomic prediction of growth and reproductive traits as assessed with 20 replicates of five-fold CV is shown in Tables 3 and 4. When predicting the traits of pigs from the Beijing population based on the GBLUP method, larger bias (0.199) was produced by ST-GBLUP_combined than by ST-GBLUP_single. Likewise, unbiasedness was close to 1 with the ST-GBLUP_single method for growth and reproductive traits when predicting the traits of pigs from the Fujian population, while ST-GBLUP_combined yielded a 0.292 larger bias than ST-GBLUP_single (Table 3). Similarly, ST-BayesC *π* also produced more bias for the combined population than for the single populations in most situations (Table 4).

In addition, prediction accuracies obtained with training on the Beijing population and validation in the Fujian population and training on the Fujian population and validation in the Beijing population using ST-GBLUP and ST-BayesC *π* were determined. Low prediction accuracies and large bias were observed in all scenarios (Additional file: Table S1), indicating that using one population to predict another is infeasible, even though the genetic backgrounds of the Beijing and Fujian populations were similar in this study.

### Comparison of the multi-trait model with the single-trait model

Both single-trait and multi-trait models with GBLUP and BayesC *π* were implemented to address the combined population in this study. Tables 3 and 4 show the accuracies and unbiasedness of genomic prediction obtained with the multi-trait and single-trait models in the same scenarios. In general, the multi-trait model showed significant superiority over the single-trait model. For the GBLUP method, as shown in Table 3, when predicting the traits of pigs from the Beijing population, the multi-trait GBLUP model (MT-GBLUP) yielded approximately 1.3% (1.2%) and 2.2% (0.8%) higher accuracies than ST-GBLUP_single (ST-GBLUP_combined) for the growth and reproductive traits, respectively. Furthermore, a small amount of bias was obtained with MT-GBLUP in most scenarios. A similar trend was found in the Fujian population. As shown in Table 3, MT-GBLUP yielded 3.2% (2.2%) and 3.0% (1.1%) higher accuracies on average for the growth and reproductive traits, respectively, than ST-GBLUP_single (ST-GBLUP_combined). The unbiasedness was close to 1 when using the MT-GBLUP methods for growth and reproductive traits in most situations. Again, the gains obtained by MT-GBLUP in the Fujian population were larger than those in the Beijing population.

For the multi-trait model with BayesC *π*, as shown in Table 4, when predicting the traits of pigs from the Beijing population, MT-BayesC *π* yielded approximately 1.3% (1.4%) and 2.5% (2.5%) higher accuracies than ST-BayesC *π* _single (ST-BayesC *π* _combined) for the growth and reproductive traits, respectively. The largest gain over ST-BayesC *π* _single and ST-BayesC *π* _combined was 2.7% and 2.9% for NBA, respectively. When predicting the traits of pigs from the Fujian population, MT-BayesC *π* obtained higher accuracies for all traits (Table 4), yielding 2.7% (2.6%) and 2.4% (2.3%) gains on average over ST-BayesC *π* _single (ST-BayesC *π* _combined) for the growth and reproductive traits, respectively. MT-BayesC *π* also produced bias as low as that of ST-BayesC *π* _single in most scenarios.

### Comparison of the GBLUP and Bayes**C*π*** methods

Figure 3 presents the accuracy of genomic prediction of growth and reproductive traits achieved by applying single-population ST-GBLUP (BayesC *π*) and MT-GBLUP (BayesC *π*) methods. When predicting the traits of pigs from the Beijing population, GBLUP yielded approximately 1.0% higher accuracy than BayesC *π* regardless of whether the single- or multi-trait model was used for AGE and a bias close to 0 with the GBLUP method in this scenario, while there was no difference in accuracy between GBLUP and BayesC *π* for BFT. For reproductive traits in the Beijing population, the accuracy of genomic prediction was increased by 1.3% on average for MT-BayesC *π* compared to MT-GBLUP, and no difference in accuracy was found between ST-GBLUP and ST-BayesC *π*. When predicting the traits of pigs from the Fujian population, GBLUP and BayesC *π* produced similar prediction accuracies for AGE; e.g., the prediction accuracies with ST-GBLUP_single and ST-BayesC *π* _single were 0.245 and 0.243, respectively, while less bias was obtained by GBLUP in this scenario. There was no difference in prediction accuracy or unbiasedness between ST-GBLUP_single and ST-BayesC *π* _single for BFT, while MT-GBLUP achieved a 1.3% higher accuracy than MT-BayesC *π*. For reproductive traits in the Fujian population, there was no difference in prediction accuracy between GBLUP and BayesC *π*, except that MT-GBLUP_combined achieved a 1.9% higher accuracy than MT-BayesC *π* _combined for TNB. Generally, GBLUP performed comparably to BayesC *π* in the single-trait and multi-trait models.

## Discussion

In this study, we investigated the accuracy of genomic prediction in two Yorkshire pig populations with similar genetic backgrounds when G × E interactions were taken into account. Our results revealed G × E interactions between the two populations for the reference growth and reproductive traits. We further explored whether directly combining populations to enlarge the reference population is efficient in improving the accuracy of genomic prediction in the presence of G × E interactions. Our results demonstrated the superiority of the multi-trait model for genomic prediction in dealing with G × E interactions, which could better accommodate the G × E interactions. Higher prediction accuracies and lower bias were obtained with the multi-trait model than with the single-trait model when implementing either GBLUP or BayesC *π* in a given situation.

G × E interactions play an important role in pig populations, and they should be considered in breeding programs to select the best animals in different environments [11, 24]. The detection of G × E interactions relies on a genetic correlation of 0.8 for one trait in different environments, the threshold suggested by Robertson [23]. Accordingly, G × E interactions between two pig populations for growth and reproductive traits (ranging from 0.618 to 0.723) were observed. Similarly, Li et al. [25] reported on the across-country genetic correlations (ranging from 0.604 to 0.726) of dairy cattle, indicating that an important G × E interaction exists between Brazilian and Nordic (or Nordic and French) populations. Hay and Roberts [26] also reported the existence of genotype-by-nutritional environment interactions for growth and carcass traits in beef cattle with genetic correlations below the threshold value of 0.8. In this study, SNP markers were used to construct the relationships (G matrix) between the two populations in order to estimate the genetic correlations, as no linked pedigree was available. Compared with a pedigree used to estimate genetic correlations, the realized relationships among individuals are captured by marker information and achieve more accurate estimates of genetic correlations. However, the number of genotyped animals was not large enough to produce a lower standard error for genetic correlations compared to that obtained by estimating heritability. We therefore used SNP markers to assess the population structure of the two pig populations, and the similar patterns of LD on each chromosome between these two populations further showed that they had similar genetic backgrounds. However, the correlation of *r*_*LD*_ values of adjacent SNPs with a mean of 0.55 indicated the insufficient consistency in LD between these two pig populations, which could be due to G × E interactions. Genetic correlations are caused by the pleiotropic action of genes and linkage between genes affecting different traits [9]. If the performance values of the same trait in different populations (environments) are regarded as different traits and are affected by different genes (that are of course pleiotropic), the phase of linkage of these genes and the consistency of their linkage (not LD) in the two populations will affect the strength of the genetic correlation and G × E interaction.

The size of the reference population is one key factor in genomic prediction [4]. Combining populations is an easy but practical way to improve the accuracy of genomic prediction, and its advantages in practice have been reported by EuroGenomics [8] and North American [2] consortiums. Similar results were shown in this study, in which higher prediction accuracies were obtained by the single-trait model for the combined population than for the single populations in most situations (Table 3), and such gains were greater for the population with a small reference size; e.g., the prediction accuracy in the combined population was 1.1% higher than that in the single population for the growth trait BFT in the Fujian population but only 0.3% higher in the Beijing population, and the number of genotyped animals in the Beijing population was much larger than that in the Fujian population (Table 2), in accordance with the features of other studies [5, 14]. However, in some situations, accuracy was not increased or slightly decreased in the combined population; e.g., the prediction accuracies for AGE in the Beijing population obtained with ST-BayesC *π* _combined and ST-BayesC *π* _single were 0.306 and 0.304, respectively (Table 4). Generally, in this study, the single-trait model exhibited only a slight gain in genomic prediction accuracy for most traits when using the combined population. In addition, the single-trait model produced more bias for the combined population than for the single populations (Tables 3 and 4). The lower improvement or slight decrease in the combined population compared with the expectation could be due to the weak consistency in LD between populations, as pointed out by Lund et al. [8].

Many studies have shown that inconsistent LD between SNPs and causative variants across populations causes disadvantages compared with using a combined population in genomic prediction [5, 6, 27, 28]. In this study, the correlation of *r*_*LD*_ values of adjacent SNPs with a mean of 0.55 indicated the insufficient consistency in LD between the Beijing and Fujian populations. Weak consistency in LD will lead to bias in SNP effect estimates when populations are simply combined with a single-trait model, further resulting in fewer improvements in accuracy and larger bias. Therefore, the effect of increasing the consistency in LD or reducing its noise on GEBVs is worth investigating. On the one hand, the consistency in LD between populations could be affected by the density of chip SNPs. De Roos et al. [29] found that markers in LD with causative variants across diverged breeds would require ~ 300,000 SNPs. Hoze et al. [30] reported that 2% higher prediction accuracies were acquired for combined small populations of dairy cattle breeds when using the 777 K panel compared to the 54 K panel, and our previous study showed that imputed whole-genome sequencing data hold the potential to increase the accuracy of genomic prediction for combined populations in pigs [7].

On the other hand, reasonable models should be explored to take advantage of the correlation of LD between populations. The single-trait model ignores the inconsistency in LD between populations, while the multi-trait model treats populations as different environments and uses covariance to consider the correlation between them, which is caused by the consistency (no matter how high or low) in LD between populations. In this study, the multi-trait model performed best in all scenarios (Tables 3 and 4). Similar results were obtained in a Brazilian Holstein population by adding data from Nordic and French Holstein populations and using a multi-trait model [25]. In addition, our results also showed that less bias was obtained with the multi-trait model compared to the single-trait model, indicating that the multi-trait model can not only use the combined information of populations to increase prediction accuracy but also reduce the impact of LD inconsistencies.

The multi-trait model and reaction norm model are two prevalent methods for handling G × E interactions in genetic evaluations. Only a few studies have explicated the advantages and disadvantages of these two kinds of methods. Falconer et al. [9] reported that G × E interactions could be detected using a multi-trait model, in which the trait values in each environment are treated as genetically distinct traits. In a limited number of environments (e.g., in this study, with two breeding farms), a multi-trait model could capture the G × E interaction between these environments. However, the computational demand of multi-trait models will increase rapidly with an increase in the number of environmental levels, as more (co)variance components will have to be estimated and convergence will become increasingly difficult. In contrast to the multi-trait model, the reaction norm model can handle a large number of or continuous environmental levels (e.g., farm, year and seasonal effects), and genetic parameters could be estimated at each environmental level [12]. However, the reaction norm model captures only part of the G × E interaction due to the limited number of environmental levels. In this study, the genomic prediction accuracies were not improved by using the reaction norm model with farm, year and seasonal effects as environmental covariates due to the limited number of environments (results not shown). Therefore, the reaction norm model could not perform well in a limited number of environments. In addition, convergence of the reaction norm model could be hindered because of the complexity of the model when the data are less informative [11]. Therefore, a multi-trait model is optimal for analyzing G × E interactions in a limited number of environments. Furthermore, the multi-trait model is more convenient in practical breeding, and it can accommodate phenotypes that are not measured in exactly the same way, e.g., different scales of deregressed proofs and the existence of G × E interactions in Chinese and Nordic Holsteins [25, 31]. Such phenotypes cannot be analyzed by a single-trait model for joint genomic prediction, while the use of a multi-trait model is plausible in such cases.

In this study, GBLUP and BayesC *π*, assuming a common (co)variance for all (GBLUP) or a proportion (BayesC *π*) of the SNP effects, were used in this study. As many studies have reported for real data [32, 33], the average prediction accuracies were not significantly different between GBLUP and BayesC *π* in the single populations and multi-trait model in this study (Fig. 3). However, BayesC *π* performed worse than GBLUP for the combined population, and no improvements in accuracy were obtained by BayesC *π* for most traits compared to that obtained with a single population. In addition, BayesC *π* produced a larger bias (Table 4). Table 5 provides the standard error of the marker effect estimated by BayesC *π* in single and combined populations. The BayesC *π* method for the combined population produced the largest standard error of the marker effect in all situations, implying larger bias when estimating genomic breeding values. A larger standard error for the marker effect might have been caused by the inconsistency in LD between the Beijing and Fujian populations. In contrast, the minimum standard error of the marker effects estimated by the multi-trait BayesC *π* model also indicated the superiority of multi-trait BayesC *π* in genomic prediction. Multi-trait BayesC *π* was first proposed by Jia and Jannink [34], with the restrictive assumption that a locus simultaneously affects all the traits or none of them. In this study, multi-trait BayesC *π* allowed a locus to affect any combination of traits, which is biologically realistic, especially in multi-trait analyses involving many traits [21]. However, it might not be possible to apply multi-trait single-step GBLUP (BayesC *π*) [35–37] in this study, as no pedigree link between the two pig populations was available and the BayesC *π* method was too computationally demanding.

**Table 5:** Mean value of the standard error of marker effects estimated by the BayesC *π* method using all genotyped animals

Trait^b^	Single-trait model			Multi-trait model
	Beijing^a^ population	Fujian^a^ population	Combined population	
AGE	0.0062	0.0079	0.0093	0.0053
BFT	0.0050	0.0069	0.0096	0.0048
NBA	0.0039	0.0047	0.0050	0.0039
TNB	0.0042	0.0051	0.0054	0.0038

^a^ Yorkshire pig populations from Beijing and Fujian with similar genetic backgrounds

^b^ AGE: days to 100 kg; BFT: backfat thickness at 100 kg; NBA: number of piglets born alive; TNB: total number of piglets born

## Conclusions

G × E interactions constitute a potential source of inefficiency in animal breeding. Ignoring possible G × E interactions could lead to a reduction in genetic gains. Our results demonstrated that directly combining populations to enlarge the reference population is not efficient in improving the accuracy of genomic prediction in the presence of G × E interactions in two Yorkshire pig populations, while the multi-trait model performed better in a limited number of environments with weak G × E interactions. This study will be helpful when leveraging G × E interactions in practical genomic selection.

## Supplementary information

**Additional file 1: Fig. S1.** Principal component analysis (PCA) of the Beijing and Fujian Yorkshire pig populations and a British Yorkshire pig population.

**Additional file 2: Table S1.** Accuracy and unbiasedness of genomic prediction of growth and reproductive traits obtained when using one population to predict another.

## Data Availability

The datasets used during the current study are available from the corresponding author on reasonable request.

## Abbreviations

G × E: Genotype-by-environment
GBLUP: Genomic best linear unbiased prediction
LD: Linkage disequilibrium
SNPs: Single nucleotide polymorphisms; Beijing and Fujian represent two Yorkshire pig populations from two Chinese pig breeding farms
AGE: Days to 100 kg
BFT: Backfat thickness at 100 kg
NBA: Number of piglets born alive
TNB: Total number of piglets born
*y*_*c*_: Corrected phenotypic values
GEBV: Genomic EBV
ST-GBLUP(BayesC *π*): Single-trait GBLUP(BayesC *π*)
MT-GBLUP(BayesC *π*): Multi-trait GBLUP(BayesC *π*)
CV: Cross validation
ST-GBLUP(BayesC *π*)_single: ST-GBLUP(BayesC *π*) based on single population
ST-GBLUP(BayesC *π*)_combined: ST-GBLUP(BayesC *π*) based on combined population
PCA: Principal component analysis
PC: Principal component
MCMC: Markov chain Monte Carlo

## References

[1] Meuwissen THE, Hayes BJ, Goddard ME (2001). Prediction of total genetic value using genome-wide dense marker maps. Genetics..

[2] VanRaden PM, Van Tassell CP, Wiggans GR, Sonstegard TS, Schnabel RD, Taylor JF (2009). Invited review: reliability of genomic predictions for north American Holstein bulls. J Dairy Sci.

[3] Zhong S, Dekkers JC, Fernando RL, Jannink J-L (2009). Factors affecting accuracy from genomic selection in populations derived from multiple inbred lines: a barley case study. Genetics..

[4] Goddard ME, Hayes BJ (2009). Mapping genes for complex traits in domestic animals and their use in breeding programmes. Nat Rev Genet.

[5] Hayes BJ, Bowman PJ, Chamberlain AC, Verbyla K, Goddard ME. Accuracy of genomic breeding values in multi-breed dairy cattle populations. Genet Sel Evol. 2009;41:51. 10.1186/1297-9686-41-51 PMC2791750 19930712

[6] Song H, Zhang J, Jiang Y, Gao H, Tang S, Mi S (2017). Genomic prediction for growth and reproduction traits in pig using an admixed reference population. J Anim Sci.

[7] Song H, Ye S, Jiang Y, Zhang Z, Zhang Q, Ding X (2019). Using imputation-based whole-genome sequencing data to improve the accuracy of genomic prediction for combined populations in pigs. Genet Sel Evol.

[8] Lund MS, Roos AP, Vries AG, Druet T, Ducrocq V, Fritz S (2011). A common reference population from four European Holstein populations increases reliability of genomic predictions. Genet Sel Evol.

[9] Falconer DS, Mackay TF, Frankham R (1996). Introduction to quantitative genetics (4th edn). Trends Genet.

[10] Meyer K (2009). Factor-analytic models for genotype× environment type problems and structured covariance matrices. Genet Sel Evol.

[11] Liu A, Su G, Hoglund J, Zhang Z, Thomasen J, Christiansen I (2019). Genotype by environment interaction for female fertility traits under conventional and organic production systems in Danish Holsteins. J Dairy Sci.

[12] Kolmodin R, Strandberg E, Madsen P, Jensen J, Jorjani H (2002). Genotype by environment interaction in Nordic dairy cattle studied using reaction norms. Acta Agriculturae Scandinavica, Section A-Animal Sci.

[13] Su G, Madsen P, Lund MS, Sorensen D, Korsgaard IR, Jensen J (2006). Bayesian analysis of the linear reaction norm model with unknown covariates. J Anim Sci.

[14] Zhou L, Ding XD, Zhang Q, Wang YC, Lund MS, Su GS. Consistency of linkage disequilibrium between Chinese and Nordic Holsteins and genomic prediction for Chinese Holsteins using a joint reference population. Genet Sel Evol. 2013;45:7. 10.1186/1297-9686-45-7 PMC3616874 23516992

[15] Madsen P, Milkevych V, Gao H, Christensen OF, Jensen J: DMU - A Package for Analyzing Multivariate Mixed Models in Quantitative Genetics and Genomics. In: Proceedings of the World Congress on Genetics Applied to Livestock Production. vol. Electronic Poster Session - Methods and Tools - Software; 2018: 525.

[16] Browning BL, Browning SR (2009). A unified approach to genotype imputation and haplotype-phase inference for large data sets of trios and unrelated individuals. Am J Hum Genet.

[17] Yang J, Lee SH, Goddard ME, Visscher PM (2011). GCTA: a tool for genome-wide complex trait analysis. Am J Hum Genet.

[18] Hill WG, Robertson A (1968). Linkage disequilibrium in finite populations. Theor Appl Genet.

[19] VanRaden PM (2008). Efficient methods to compute genomic predictions. J Dairy Sci.

[20] Garrick D, Dekkers J, Fernando R (2014). The evolution of methodologies for genomic prediction. Livest Sci.

[21] Cheng H, Kizilkaya K, Zeng J, Garrick D, Fernando R (2018). Genomic prediction from multiple-trait Bayesian regression methods using mixture priors. Genetics..

[22] Cheng H, Fernando R, Garrick D: JWAS: Julia implementation of Whole-genome Analyses Software. In: Proceedings of the World Congress on Genetics Applied to Livestock Production. vol. Methods and Tools - Software; 2018: 859.

[23] Robertson (1959). The sampling variance of the genetic correlation coefficient. Biometrics.

[24] Zhang Z, Kargo M, Liu AX, Thomasen JR, Pao YC, Su GS (2019). Genotype-by-environment interaction of fertility traits in Danish Holstein cattle using a single-step genomic reaction norm model. Heredity..

[25] Li X, Lund MS, Zhang Q, Costa CN, Ducrocq V, Su G (2016). Short communication: improving accuracy of predicting breeding values in Brazilian Holstein population by adding data from Nordic and French Holstein populations. J Dairy Sci.

[26] Hay EH, Roberts A (2018). Genotype× prenatal and post-weaning nutritional environment interaction in a composite beef cattle breed using reaction norms and a multi-trait model. J Anim Sci.

[27] Erbe M, Hayes BJ, Matukumalli LK, Goswami S, Bowman PJ, Reich CM (2012). Improving accuracy of genomic predictions within and between dairy cattle breeds with imputed high-density single nucleotide polymorphism panels. J Dairy Sci.

[28] van den Berg I, Bowman PJ, MacLeod IM, Hayes BJ, Wang T, Bolormaa S (2017). Multi-breed genomic prediction using Bayes R with sequence data and dropping variants with a small effect. Genet Sel Evol.

[29] De Roos A, Hayes BJ, Spelman R, Goddard ME (2008). Linkage disequilibrium and persistence of phase in Holstein–Friesian. Jersey Angus Cattle Genetics.

[30] Hoze C, Fritz S, Phocas F, Boichard D, Ducrocq V, Croiseau P (2014). Efficiency of multi-breed genomic selection for dairy cattle breeds with different sizes of reference population. J Dairy Sci.

[31] Li XJ, Lund MS, Janss L, Wang CL, Ding XD, Zhang Q, et al. The patterns of genomic variances and covariances across genome for milk production traits between Chinese and Nordic Holstein populations. BMC Genet. 2017;18:26. 10.1186/s12863-017-0491-9 PMC5353867 28298201

[32] Hayes BJ, Bowman PJ, Chamberlain AJ, Goddard ME (2009). Invited review: genomic selection in dairy cattle: progress and challenges. J Dairy Sci.

[33] Zhang CY, Kemp RA, Stothard P, Wang ZQ, Boddicker N, Krivushin K, et al. Genomic evaluation of feed efficiency component traits in Duroc pigs using 80K, 650K and whole-genome sequence variants. Genet Sel Evol. 2018;50:14. 10.1186/s12711-018-0387-9 PMC5889553 29625549

[34] Jia Y, Jannink JL (2012). Multiple-Trait Genomic Selection Methods Increase Genetic Value Prediction Accuracy. Genetics.

[35] Misztal I, Legarra A, Aguilar I (2009). Computing procedures for genetic evaluation including phenotypic, full pedigree, and genomic information. J Dairy Sci.

[36] Christensen OF, Lund MS. Genomic prediction when some animals are not genotyped. Genet Sel Evol. 2010;42:2. 10.1186/1297-9686-42-2 PMC2834608 20105297

[37] Fernando RL, Dekkers JC, Garrick DJ (2014). A class of Bayesian methods to combine large numbers of genotyped and non-genotyped animals for whole-genome analyses. Genet Sel Evol.

