# Supplementary figures and images for: The superiority of multi-trait models with genotype-by-environment interactions in a limited number of environments for genomic prediction in pigs
Source: J Anim Sci Biotechnol. 2020 Aug 19;11:88. doi: 10.1186/s40104-020-00493-8 (PMC7507970; doi:10.1186/s40104-020-00493-8)

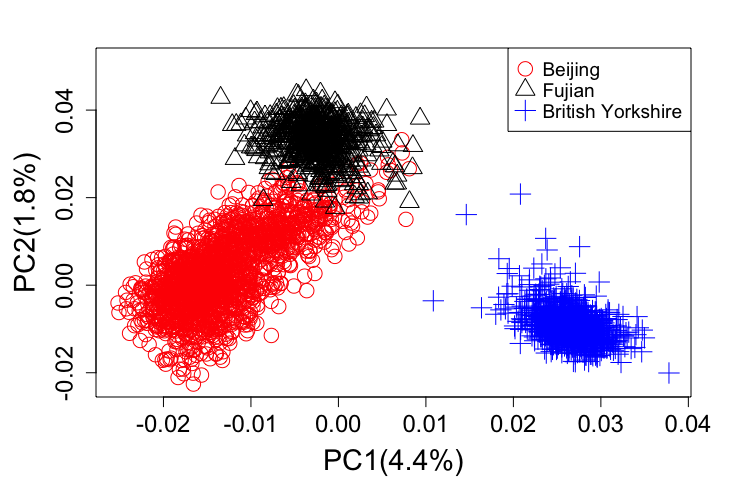

Supplement: Supplementary file 1 — Additional file 1: Fig. S1. Principal component analysis (PCA) of the Beijing and Fujian Yorkshire pig populations and a British Yorkshire pig population. [file 40104_2020_493_MOESM1_ESM.tiff]
