# Supplementary material for: The superiority of multi-trait models with genotype-by-environment interactions in a limited number of environments for genomic prediction in pigs
Source: J Anim Sci Biotechnol. 2020 Aug 19;11:88. doi: 10.1186/s40104-020-00493-8 (PMC7507970; doi:10.1186/s40104-020-00493-8)
Supplement: Supplementary file 2 — Additional file 2: Table S1. Accuracy and unbiasedness of genomic prediction of growth and reproductive traits obtained when using one population to predict another. [file 40104_2020_493_MOESM2_ESM.docx]

**Table S1** **Accuracy and unbiasedness of genomic prediction of growth and reproductive traits obtained when using one population to predict another.**

| **Reference population^1^** | **Validation**  **population^1^** | **Method** | **Measurement^2^** | **AGE^3^** | **BFT^3^** | **NBA^3^** | **TNB^3^** |
| --- | --- | --- | --- | --- | --- | --- | --- |
| Beijing | Fujian | GBLUP | Accuracy  Unbiasedness | 0.108  1.315 | 0.118  1.290 | 0.080  1.214 | 0.100  1.322 |
|  |  | BayesC$\pi$ | Accuracy  Unbiasedness | 0.103  1.337 | 0.109  1.301 | 0.070  1.325 | 0.104  1.418 |
| Fujian | Beijing | GBLUP | Accuracy  Unbiasedness | 0.076  0.742 | 0.103  0.566 | 0.060  0.794 | 0.097  0.765 |
|  |  | BayesC$\pi$ | Accuracy  Unbiasedness | 0.081  0.637 | 0.100  0.457 | 0.064  0.583 | 0.096  0.734 |

^1^ Yorkshire pig populations from Beijing and Fujian with similar genetic backgrounds.

^2^ Accuracy: the correlation between GEBVs and corrected phenotypic values in the validation population; Unbiasedness: the regression of corrected phenotypic values on GEBVs.

^3^ AGE: days to 100 kg; BFT: backfat thickness at 100 kg; NBA: number of piglets born alive; TNB: total number of piglets born.
